# Intratumoral microbiome: a key regulator and novel therapeutic target for chemoresistance in pancreatic cancer

**DOI:** 10.3389/fcimb.2026.1860523

**Published:** 2026-06-19

**Authors:** Yunpeng Wang, Jiao Wu

**Affiliations:** 1 Department of Cardiology, Mianyang Central Hospital, School of Medicine, University of Electronic Science and Technology of China, Mianyang, China; 2 Department of Gastroenterology, Mianyang Central Hospital, School of Medicine, University of Electronic Science and Technology of China, Mianyang, China

**Keywords:** chemotherapy resistance, intratumoral microbiome, pancreatic cancer, therapeutic target, tumor microenvironment

## Abstract

Pancreatic cancer is a highly lethal gastrointestinal malignancy with chemotherapy resistance as a major obstacle to improving prognosis. Emerging evidence indicates that the intratumoral microbiome is closely implicated in the development, progression, and therapeutic response of pancreatic cancer. The intratumoral microbiome of pancreatic cancer is mainly composed of *Proteobacteria* and *Firmicutes*, and its composition is significantly correlated with patient survival. Intratumoral microbes drive tumor progression by remodeling the immune microenvironment, inducing DNA damage, and activating oncogenic signaling pathways. Meanwhile, they exacerbate chemotherapy resistance via multiple mechanisms, including remodeling the extracellular matrix, establishing an immunosuppressive microenvironment, and metabolically inactivating chemotherapeutic agents. This review systematically summarizes the community composition of the intratumoral microbiome in pancreatic cancer, its regulatory effects, and underlying mechanisms on chemotherapy resistance, as well as the latest advances in targeted therapeutic strategies.

## Introduction

Data from the American Cancer Society, derived from 2021 incidence data of the Central Cancer Registry and 2022 mortality data from the National Center for Health Statistics, projected that pancreatic cancer will become the third leading cause of cancer-related mortality in the United States by 2025 (Siegel et al., 2025) and rise to the second leading cause by 2030 (Rahib et al., 2021). The incidence-to-mortality ratio of pancreatic cancer in the United States is 1.3:1. In China, although pancreatic cancer ranks sixth among all cancer-related deaths, its incidence-to-mortality ratio is 1.12:1 (Han et al., 2024). Notably, pancreatic cancer has the highest incidence-to-mortality ratio across all malignancies in both countries. A major obstacle to the treatment of pancreatic cancer is its insidious onset. Due to the absence of specific early symptoms and its inherent invasive nature, most patients are diagnosed at an advanced stage with established distant metastasis (Stoop et al., 2025). Accordingly, the median overall survival of patients with untreated advanced pancreatic cancer remains poor, at approximately 4 months (Mackay et al., 2024; Siegel et al., 2024). Over the past three decades, advances in precision oncology, minimally invasive surgery, and standardized multidisciplinary care have yielded modest survival benefits: the 5-year overall survival rate has increased from 4% to 13% (Siegel et al., 2024). Despite such gradual improvements, pancreatic cancer still maintains one of the highest mortality-to-incidence ratios of all human cancers.

Surgical resection combined with systemic chemotherapy constitutes the cornerstone of curative-intent treatment for pancreatic ductal adenocarcinoma (PDAC) (hereinafter referred to collectively as pancreatic cancer), yet only 15–20% of patients present with resectable disease at diagnosis (Boubaddi et al., 2025). Therefore, palliative chemotherapy remains the standard of care for patients with metastatic or locally advanced unresectable pancreatic cancer (Zhao et al., 2025). Among first-line regimens, FOLFIRINOX (oxaliplatin, irinotecan, leucovorin, fluorouracil) and gemcitabine plus albumin-bound paclitaxel (gem-nabP) are the most widely used and guideline-endorsed options (Yang et al., 2020). The median overall survival and overall survival rate for the FOLFIRINOX regimen are approximately 11 months and 48%. The median survival and overall survival rates for the gemcitabine-albumin-bound paclitaxel regimen are approximately 9 months and 35% (Stoop et al., 2025). These modest outcomes highlight that even with modern first-line chemotherapy, survival for metastatic pancreatic cancer remains unsatisfactorily low, reflecting widespread intrinsic and acquired chemoresistance. A major contributor to treatment failure is the dense, profibrogenic stroma, which elevates intratumoral pressure, creates a physical barrier, and impairs drug penetration into tumor nests (Hosein et al., 2020). Equally critical is the development of chemotherapy resistance, driven by cancer cell-intrinsic alterations and by the pancreatic cancer tumor microenvironment (TME), including pancreatic stellate cells (PSCs), cancer-associated fibroblasts (CAFs), and tumor-associated macrophages (TAMs) (Wu et al., 2026). Epigenetic reprogramming, apoptotic dysregulation, and stromal cell-mediated immunosuppression further reinforce resistance and limit durable responses (Cruz et al., 2024). Unfortunately, most therapies targeting these canonical resistance mechanisms have failed in the clinic, due to compensatory pathway rewiring, metabolic reprogramming, and a lack of highly selective, stroma-penetrant inhibitors (de Castilhos et al., 2024). There is therefore an urgent unmet need to identify novel, actionable determinants of treatment efficacy and to develop rational combinatorial strategies to improve pancreatic cancer outcomes.

Emerging evidence indicates that the microbiome is intricately linked to pancreatic cancer oncogenesis, progression, and therapeutic response, and may serve as a biomarker for early detection, prognosis, and chemoresistance (Tabrizi et al., 2024). The intratumoral microbiome may possess unique adaptive capabilities that enable survival within the tumor microenvironment, potentially altering tumor response to chemotherapy (Ernst et al., 1999; Ji et al., 2023). Notably, recent clinical data demonstrate that standard antibiotic regimens do not significantly alter the overall composition or diversity (α-diversity or β-diversity) of the pancreatic cancer intratumoral microbiome, implying the relative stability and potential targetability of this niche (Abe et al., 2024). Accordingly, investigating the functional impact and molecular mechanisms of the intratumoral microbiome in pancreatic cancer progression and chemoresistance, as well as developing microbiome-modulating adjuvant therapies, holds substantial translational and clinical significance.

## Intratumoral microbiome features in pancreatic cancer tissues

The microbiome refers to the collective community of microorganisms (including bacteria, archaea, fungi, and viruses) and their genetic material that colonize a specific habitat (Kumar et al., 2026). This complex ecosystem is closely associated with systemic diseases affecting multiple organs. While the gut is recognized as the primary habitat for the majority of host-associated microbiota, advances in genomic sequencing technology in recent years have uncovered the presence of microbiota within solid tumor tissues—an assemblage specifically termed the tumor microbiome (Human Microbiome Project, 2012; Nejman et al., 2020). Geller et al. were the first to identify bacteria in human pancreatic cancer tissue, with the predominant bacterial class being *Gammaproteobacteria*. In their study, 16S ribosomal DNA (rDNA) was collected from pancreatic cancer samples of 113 patients and normal pancreatic tissue from 20 organ donors. Results showed that only 15% of normal pancreatic tissues contained detectable bacteria, whereas this proportion reached 76% in pancreatic cancer samples (Geller et al., 2017). However, early studies on pancreatic cancer microbial detection faced skepticism due to limitations such as insufficient sample sizes and inadequate decontamination procedures, which raised concerns about result reliability.

Two key pathways have been proposed to explain microbial colonization of the pancreatic tumor microenvironment: First, the pancreas is endowed with a rich vascular network, enabling microorganisms to spread hematogenously and establish colonization (Shi et al., 2020). Second, the pancreas connects to the intestines via the pancreatic duct, providing a direct route for gut microbiota to migrate and colonize pancreatic tissues (Jiang et al., 2023). With continuous advancements in detection technology and computational analysis methods, researchers have gained increasingly clear insights into the characteristics of the intratumoral microbiome in pancreatic cancer. Shohei Abe et al. employed quantitative polymerase chain reaction (qPCR) and *in situ* hybridization techniques to screen 162 paraffin-embedded pancreatic cancer tissue samples, identifying 52 specimens containing detectable intratumoral microbiota. Subsequent 16S rRNA gene sequencing of these 52 positive samples and matched non-tumor controls revealed that PDAC tissues were highly enriched in the bacterial phyla *Proteobacteria* and *Firmicutes*. Additionally, the presence of three specific genera—*Bacteroides*, *Lactobacillus*, and *Peptostreptococcus* was significantly associated with poor prognosis in PDAC patients (Abe et al., 2024). Jingze Leng et al. conducted a linear discriminant analysis effect size (LEfSe) study using pancreatic cancer samples from the The Cancer Genome Atlas (TCGA) database. They identified significant differences in the abundance of seven microbial taxa between patients with short survival and long survival: the phylum *Actinobacteria*, family *Thermomonadaceae*, class *Nitrosomonadales*, family *Spirochaetaceae*, family *Fishellaceae*, and order *Sulfurimonadales*. Notably, *Actinobacteria* showed significant differences at multiple taxonomic levels (family, order, and genus). Among genera, *Actinomadura*, *Pseudoxanthomonas*, and *Neorickettsia* were more abundant in short-survival patients, while *Mediterraneibacter*, *Cedecea*, *Hafnia*, *Azotobacter*, *Stenotrophomonas*, *Porphyromonas*, and *Desulfotalea* were more prevalent in long-survival patients (Leng et al., 2024). Ferga C Gleeso et al. analyzed 18 pancreatic exocrine tumor specimens collected via endoscopic ultrasound. 16S rRNA sequencing detected bacterial DNA in all specimens. Dominant phyla included *Proteobacteria*, *Bacteroidetes*, *Firmicutes*, and *Actinobacteria*. *Gammaproteobacteria*, *Fusobacterium*, and *Bifidobacterium* were detected in 88%, 29%, and 12% of tumors, respectively. However, no significant differences in α-diversity (within-sample microbial diversity) or β-diversity (between-sample microbial community variation) metrics were observed between anatomical locations or among major phyla and genera (Gleeson et al., 2022).

In a preclinical study, Nina Pfisterer et al. applied LEfSe analysis to mouse pancreatic cancer tissues and healthy pancreatic controls. Results showed that healthy pancreatic tissues had a higher abundance of *Lactobacillales* (particularly the species *Lactobacillus fermentum* and *Streptococcus* spp.) compared to pancreatic cancer samples, while pancreatic cancer tissues were characterized by enrichment of *Gammaproteobacteria* (particularly the *Enterobacteriaceae* family containing *Shigella* and *Citrobacter*) (Pfisterer et al., 2023). Notably, mouse pancreatic cancer samples exhibited higher relative abundances of bacterial genera and species compared to healthy pancreatic samples, with compositional features resembling the microbiome of human PDAC.

A critical limitation of previous studies is that most used non-tumor tissue adjacent to pancreatic cancer as controls, which may not fully represent healthy pancreatic microbiota. To address this gap, Francesca Tavano et al. employed healthy human pancreatic tissue as a control group to clarify microbial differences between normal and pancreatic cancer tissues. Their findings revealed reduced abundance of bacteria with potential probiotic effects (*Jeotgalicoccus*) and anti-cancer activity (*Acinetobacter guillouiae*) in PDAC patients. Additionally, Bacteria involved in immune homeostasis and tumor progression inhibition (*Streptococcus salivarius, Sphingomonas*) were decreased, while bacteria associated with tumorigenesis and progression (*Methylobacterium methylorubrum, g_Delftia*) were increased in tumor samples (Tavano et al., 2025).

To further evaluate microbial changes during pancreatic cancer progression, Wei Wang et al. collected 362 fresh pancreatic tissue samples from multiple hospitals, including malignant (PDAC), precancerous lesions (grade 1 and 2 intraductal papillary mucinous neoplasms), and benign (serous cystadenoma) tissues. They assessed species-level average relative abundance differences across benign, precancerous, and malignant pancreatic tumors. Findings suggest that survival rates in PDAC patients may be influenced by 11 specific genera: *Blautia, Faecalibacterium, Comamonas, Coprococcus, Erysipelatoclostridium, Sulfuritalea, Thermomonas, Phenylobacterium, Raoultibacter, Erysipelothrix*, and *Thiothrix* (Wang et al., 2024).

Additionally, multiple studies have analyzed the microbial community profiles within tumors of Chinese pancreatic cancer patients. Both α-diversity and β-diversity analyses revealed significant differences between tumor tissue and adjacent non-cancerous tissue, with bacterial relative abundance generally higher in tumor tissue. The predominant microbial phyla remained *Proteobacteria*, *Firmicutes*, *Actinobacteria*, and *Bacteroidetes*, though *Proteobacteria*, *Firmicutes*, and *Actinobacteria* were widely present in both tumor and adjacent normal tissues. Notably, *Pseudomonas* bacteria exhibited high abundance in the pancreatic cancer microenvironment and were significantly associated with prolonged overall survival in patients (Huang et al., 2022; Gao et al., 2024).

In summary, cumulative evidence from multiple studies indicates that regardless of differences in pancreatic cancer sample types, geographical regions (countries), or host species (human vs. mouse), distinct microbial differences exist between pancreatic cancer tissues and normal pancreatic tissues. The vast majority of research results are consistent in identifying *Proteobacteria* and *Firmicutes* as significantly enriched bacterial phyla in pancreatic cancer. Furthermore, the intratumoral microbiome is closely associated with patient survival outcomes. Consequently, this complex microbial community holds significant potential as a biomarker for the early diagnosis and prognosis of pancreatic tumors (**Table 1**).

**Table 1:** Intratumoral microbiome characteristics in different pancreatic tissues.

Research subject / site	Core microbiota characteristics	Key differences / correlations	References
Normal pancreatic tissue (human)	Only 15% harbored bacteria. Jeotgalicoccus, Acinetobacter guillouiae, Streptococcus salivarius, and Sphingomonas are enriched.	Significant differences were observed between pancreatic cancer tissues and normal tissues in terms of microbial presence rates and composition.	(Shi et al., 2020)
Pancreatic cancer tissue (human)	76% contain bacteria. The dominant bacterial phyla are Proteobacteria, Firmicutes, Bacteroidetes, and Actinobacteria.	Bacteroides, Lactobacillus, and Peptostreptococcus are associated with poor prognosis. Certain microbial communities correlate with survival duration (e.g., Actinomadura is associated with short survival duration).	(Geller et al., 2017; Gleeson et al., 2022; Abe et al., 2024; Leng et al., 2024)
Pancreatic cancer tissue (mouse)	Characterized by the γ-Proteobacteria class (Enterobacteriaceae family, including Shigella and Citrobacter); relative abundance of bacterial genera and species is higher than in healthy pancreas.	Similar to the microbial composition characteristics of human PDAC.	(Pfisterer et al., 2023)
Tumor tissue from chinese pancreatic cancer patients	α and β diversity differed significantly between tumor and adjacent normal tissues. Bacterial relative abundance was higher, with dominant phyla being Proteobacteria, Firmicutes, Actinobacteria, and Bacteroidetes.	High abundance of Pseudomonas species is associated with prolonged overall survival.	(Huang et al., 2022; Gao et al., 2024)
Pancreatic tumor tissue at different stages (benign → precancerous → malignant)	The abundance of 11 bacterial genera, including Blautia and Faecalibacterium, may influence survival rates in PDAC patients.	Microbiome composition exhibits differences with tumor progression.	(Wang et al., 2024)

## The impact of intratumoral microbiome on pancreatic cancer progression

Although multiple studies have identified microbial communities within the pancreatic cancer microenvironment and demonstrated their close association with pancreatic cancer diagnosis and prognosis, research on specific bacterial populations and pancreatic cancer progression remains limited. *Fusobacterium nucleatum*, a bacterium belonging to the phylum *Firmicutes*, induces increased secretion of cytokines such as GM-CSF, CXCL1, IL-8, and MIP-3α from both normal pancreatic epithelial cells and PDAC cells. These cytokines significantly enhance PDAC cell proliferation, migration activity, and invasiveness (Udayasuryan et al., 2022). Furthermore, *Fusobacterium nucleatum* activates the CXCL1-CXCR2 signaling pathway to form an immunosuppressive tumor microenvironment; blocking this pathway effectively inhibits tumor growth (Hayashi et al., 2023). Intratumoral fungal communities secrete IL-33, which recruits and activates TH2 cells and innate lymphoid cells 2 (ILC2), leading to the release of inflammatory factors that promote tumor growth (Alam et al., 2022). These findings reveal that the intratumoral microbiome can promote pancreatic cancer progression by modulating the tumor immune microenvironment, consistent with previous studies. For instance, the abundance of bacteria associated with poor prognosis showed significant negative correlations with the number of CD4^+^, CD8^+^, and CD45RO^+^ T cells in PDAC tissues (Abe et al., 2024). Bacterial abundance with potential beneficial and anti-cancer activities is markedly reduced in PDAC tissues, alongside diminished microbial functions involved in maintaining immune homeostasis (Tavano et al., 2025). Previous studies indicate that *Escherichia coli* (*E. coli*) promotes pancreatic cancer progression by suppressing immune responses (Luo et al., 2020). *Escherichia coli* belongs to the *Proteobacteria* phylum, which is a microorganism abundantly infiltrating the pancreatic cancer tumor microenvironment. Furthermore, the microbiome within pancreatic tumors is partially colonized by migrating gut microbiota. Therefore, it is reasonable to hypothesize that relevant gut microbiota migrate to the pancreas, thereby modulating immune responses and promoting pancreatic cancer progression.

Beyond modulating immune responses to influence pancreatic cancer progression, numerous pathogenic microorganisms have evolved compounds capable of inducing DNA damage, cell cycle arrest, and genetic instability. These compounds accumulate within the tumor microenvironment, causing DNA damage, chromosomal instability, and reduced cellular DNA repair capacity. Ultimately, this leads to dysregulated cell growth and tumorigenesis (Jiang et al., 2023). *Escherichia coli* B2 strains secrete colibactin, which induces genomic instability and DNA double-strand breaks, thereby promoting carcinogenesis. Colibactin is the most potent DNA-damaging metabolite secreted by the intratumoral microbiome (Zhang and Sun, 2024; Chu et al., 2025). While direct evidence linking intratumoral microbiome-induced DNA damage to pancreatic cancer progression is still emerging, the consistent detection of *Escherichia coli* in pancreatic tumor tissues supports the hypothesis that this mechanism contributes to pancreatic cancer development.

Similarly, the intratumoral microbiome promotes pancreatic cancer progression by activating oncogenic signaling pathways. *Fusobacterium nucleatum*, a key intratumoral bacterium in pancreatic cancer, produces the adhesive FadA protein that binds to E-cadherin, thereby activating β-catenin signaling to drive tumor progression (Chattopadhyay et al., 2019). Drawing parallels to conserved oncogenic mechanisms across tumor types, intratumoral microbiotas are known to modulate pathways including PI3K, JAK-STAT, WNT/β-catenin, and ERK— all of which play critical roles in pancreatic cancer cell proliferation, survival, and metastasis (Katz et al., 2011; Garrett, 2015; Wong-Rolle et al., 2021). These findings collectively indicate that oncogenic pathway activation is a conserved mechanism through which intratumoral microorganisms promote pancreatic cancer progression.

**Figure 1:**
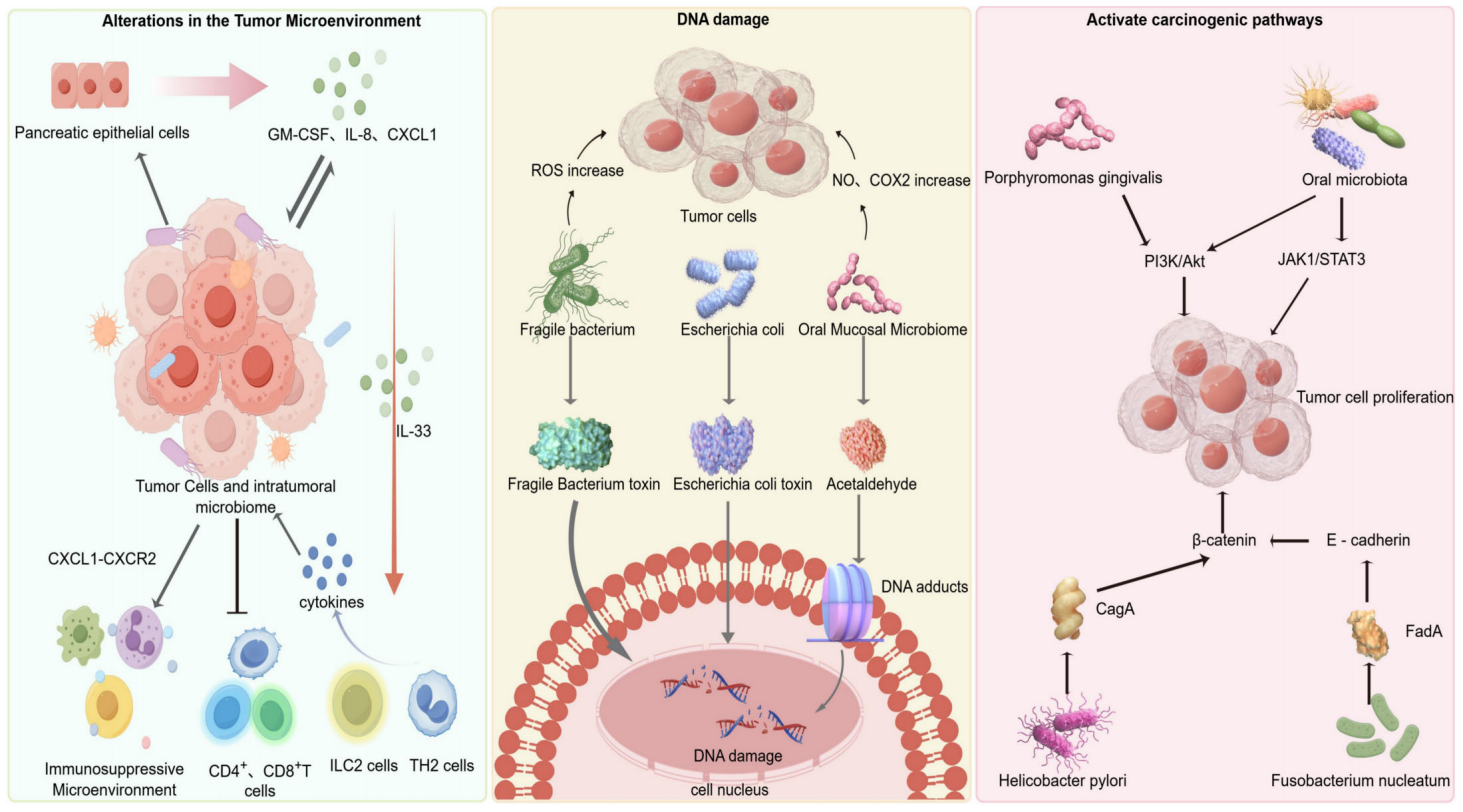
Intratumoral microbiome promotes pancreatic cancer progression through three mechanisms: Modulating the tumor immune microenvironment by regulating immune cells (e.g., T cells, macrophages) and cytokines to shape an immunosuppressive phenotype. Inducing host cell DNA damage by secreting specific metabolites that directly or indirectly cause host cell DNA damage, driving genomic instability. And activating oncogenic signaling pathways like PI3K/AKT and JAK1/STAT3 to accelerate tumor cell proliferation and invasion.

In conclusion, alterations in the tumor immune microenvironment, DNA damage induced by secreted metabolites, and activation of oncogenic pathways remain the three primary mechanisms by which the intratumoral microbiome promotes tumor progression (**Figure 1**).

## The influence of intratumoral microbiome on chemotherapy resistance in pancreatic cancer and its regulatory mechanisms

**Figure 2:**
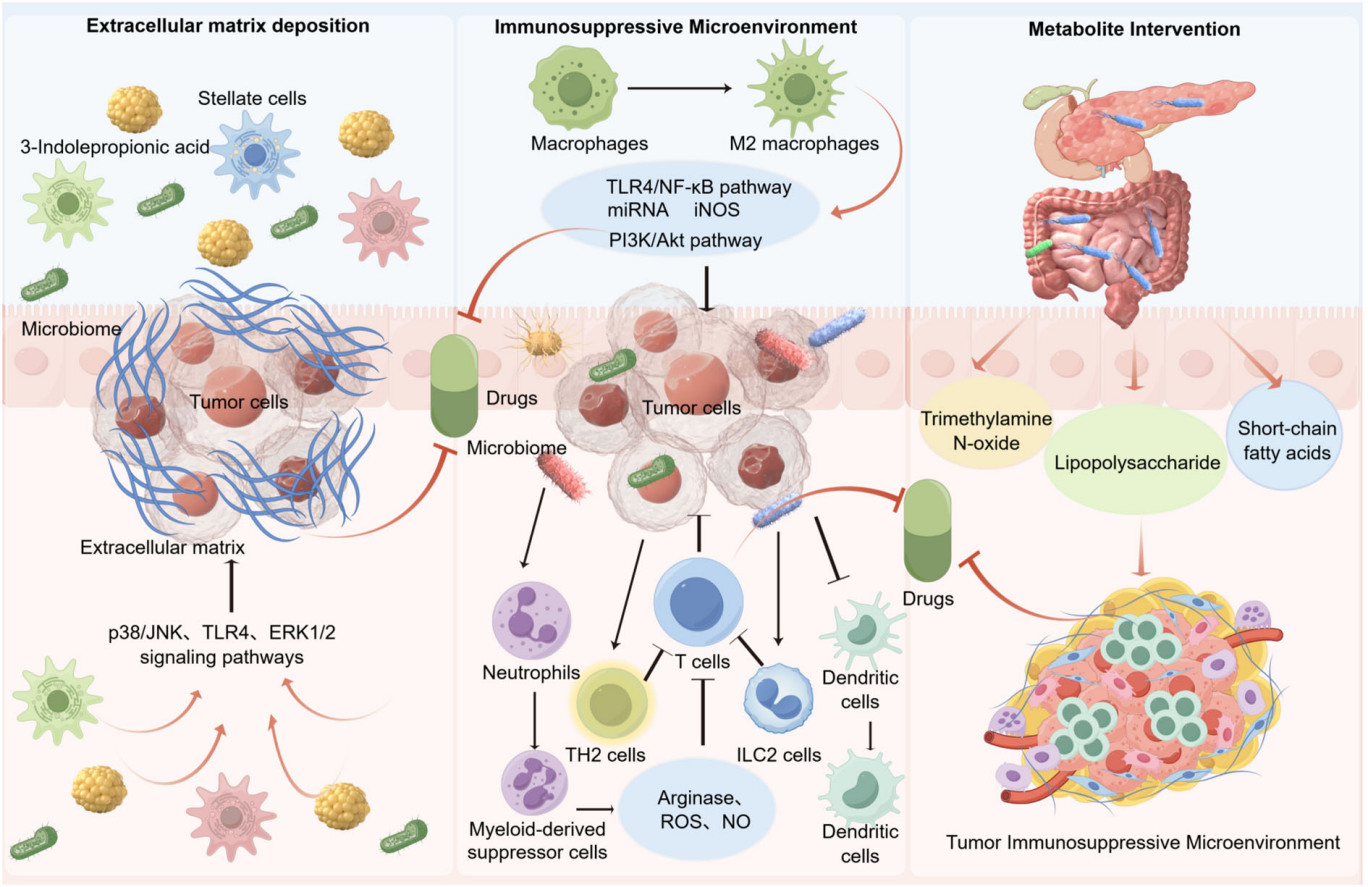
Intratumoral microbiome can reduce tumor cell sensitivity to chemotherapeutic drugs through two core pathways: First, by modulating the activation state of stellate cells, activating downstream signaling pathways such as p38 and ERK, thereby driving abnormal extracellular matrix deposition to form a physical barrier that impedes chemotherapeutic drug penetration. Second, by modulating the phenotype and function of immune cells, including macrophages, T cells, and neutrophils, and secreting active metabolites such as short-chain fatty acids, it reshapes the locally immunosuppressive tumor microenvironment, thereby weakening chemotherapy-induced antitumor immune responses.

Although chemotherapy remains the primary treatment for pancreatic cancer, it cannot completely suppress tumor progression and metastasis. Chemotherapy resistance urgently requires resolution. The intratumoral microbiome and its metabolites enhance pancreatic tumors’ resistance to chemotherapy (Zitvogel et al., 2018). In recent years, significant breakthroughs have been achieved in research combining microbiome therapies with chemotherapy drugs for cancer treatment. When co-cultured with multiple chemotherapeutic agents such as doxorubicin, gemcitabine, and verapamil *in vitro*, *Escherichia coli* significantly reduced the cytotoxicity of these drugs (Lehouritis et al., 2015). *E. coli* is one of the microbial communities present within pancreatic tumors (Li et al., 2022). Targeting the intratumoral microbiome within pancreatic tumors can reduce chemotherapy resistance, enhance drug sensitivity, and ultimately improve treatment efficacy. Currently, little is known about the regulatory mechanisms by which the intratumoral microbiome modulates chemotherapy drug resistance. Considering that the tumor microenvironment is a key mediator of treatment resistance and tumor progression, and that the microbiota significantly regulates the tumor microenvironment, the following analysis examines the mechanisms by which the intratumoral microbiome influences chemotherapy resistance in pancreatic cancer from the perspective of the tumor microenvironment (**Figure 2**).

### The role of intratumoral microbiota in regulating pancreatic cancer immunosuppressive microenvironment

Intratumoral microbiome can diminish the cytotoxic effects of chemotherapy drugs by shaping immunosuppressive microenvironments. Recent evidence indicates that microbes can influence immunotherapy responses, regulate immune checkpoints, and facilitate cancer cell escape from the immune system (Nista et al., 2023). Tumor-associated macrophages constitute a critical component of the pancreatic cancer microenvironment, representing one of the earliest infiltrating cell types in tumorigenesis. They play pivotal roles in pancreatic cancer development, metastasis, angiogenesis, immune regulation, and chemotherapy resistance (Zhou et al., 2023). Altered macrophage polarization represents a major mechanism of chemotherapy resistance in pancreatic cancer. For instance, M2-polarized macrophages generate extracellular vesicles highly expressing miR-222-3p. These miR-222-3p-containing vesicles enhance gemcitabine resistance in pancreatic cancer by suppressing TSC1 and activating the PI3K/AKT/mTOR pathway (Guo et al., 2023). Multiple microorganisms have been shown to be closely associated with macrophage M2 polarization. *Fusobacterium nucleatum* promotes macrophage infiltration by activating the miR-1322/CCL20 axis while inducing M2 macrophage polarization, thereby enhancing CRC metastasis (Xu et al., 2021). *Fusobacterium nucleatum* also induces macrophage M2 polarization by activating the TLR4/NF-κB/S100A9 pathway, promoting colon cancer development (Hu et al., 2021). Furthermore, excessive *Propionibacterium acnes* in gastric cancer patients promotes macrophage M2 polarization via TLR4/PI3K/Akt signaling (Li et al., 2021). *Staphylococcus aureus* biofilms can reduce iNOS expression and drive M2 macrophage polarization (Thurlow et al., 2011). Antibiotic-induced gut dysbiosis shifts pulmonary macrophage polarization toward an alternatively activated M2 phenotype (Kim et al., 2014). Therefore, targeting M2-polarized TAMs may represent a therapeutic option for overcoming chemotherapy resistance in pancreatic cancer.

Additionally, *Fusobacterium nucleatum* suppresses the proliferation and cytotoxic function of CD8^+^ T cells while promoting angiogenesis and tumor cell invasion and metastasis, thereby reducing the efficacy of chemotherapy drugs in eliminating tumor cells (Udayasuryan et al., 2022). *Fusobacterium nucleatum* activates the CXCL1-CXCR2 signaling pathway to recruit neutrophils into the tumor microenvironment. These neutrophils are induced into myeloid-derived suppressor cells (MDSCs) with an immunosuppressive phenotype. MDSCs directly suppress T cell-mediated antitumor immune responses by producing substances such as cathepsins, reactive oxygen species, and nitric oxide, enabling tumor cells to evade immune surveillance and chemotherapy drug attacks (Hayashi et al., 2023). Certain intratumoral bacteria also influence the immune microenvironment by modulating dendritic cells (DCs) function. Under normal conditions, DCs as professional antigen-presenting cells effectively capture, process, and present tumor antigens to activate naive T cells. However, certain intratumoral bacteria, such as *Escherichia coli*, secrete lipopolysaccharides (LPS) that continuously stimulate DCs into a “tolerant” state. This reduces their antigen-presenting capacity and decreases the expression of co-stimulatory molecules (e.g., CD80, CD86), thereby failing to effectively activate anti-tumor immune responses and indirectly promoting tumor cell resistance to chemotherapy (Luo et al., 2020). IL-33 secreted by intratumoral fungal communities not only recruits and activates TH2 cells and ILC2s but may also exacerbate the immunosuppressive microenvironment through cytokines like IL-4 and IL-13 released by these cells. This suppresses CD8^+^ T cell infiltration and function, thereby impairing chemotherapy efficacy (Alam et al., 2022). These findings indicate that the intratumoral microbiome regulates the formation of the immunosuppressive microenvironment through multiple pathways, representing a key mechanism underlying chemotherapy resistance in pancreatic cancer.

Of note, PDAC is widely recognized as an immunologically “cold” tumor characterized by low CD8+ T-cell infiltration, abundant immunosuppressive cells, and poor responsiveness to immune checkpoint inhibitors (Ullman et al., 2022). The profoundly immunosuppressive tumor microenvironment is a primary cause of both chemotherapy resistance and immunotherapy failure in PDAC (Sherman and Beatty, 2023). Accumulating evidence indicates that the intratumoral microbiome actively contributes to maintaining the immune-cold phenotype, thereby reinforcing immune evasion and multi-modal treatment resistance (Wang et al., 2025; Silver et al., 2026). Collectively, the intratumoral microbiome functions as a critical driver that sustains the immune-cold state of PDAC by suppressing T-cell function, inducing M2 macrophage polarization, and expanding MDSCs. This microbial−mediated immunosuppression not only reduces chemotherapy efficacy but also directly explains the inherent resistance of PDAC to immune checkpoint blockade. Targeting the intratumoral microbiome thus represents a promising strategy to convert cold tumors into hot tumors, restore anti−tumor immunity, and overcome both chemoresistance and immunotherapy resistance in PDAC.

### The role of intratumoral microbiota derived metabolites in regulating chemoresistance in pancreatic cancer

The gut microbiota-derived tryptophan metabolite indole-3-acetic acid (3-IAA) serves as a key amplifier of chemotherapy response in PDAC patients. During FOLFIRINOX treatment, elevated concentrations of 3-IAA and myeloperoxidase increase reactive oxygen species accumulation, impairing cancer cell stress adaptation and ultimately reducing PDAC cell proliferation (Tintelnot et al., 2023). Moreover, microbial metabolites can modulate immune responses. The microbial metabolite trimethylamine N-oxide (TMAO) induces immunogenicity in TAMs, promotes effector T cell activity, transforms the tumor microenvironment into an immunologically activated state, and sensitizes PDAC to checkpoint immunotherapy, thereby inhibiting pancreatic cancer progression (Mirji et al., 2022). Short-chain fatty acids (SCFAs), metabolites secreted by microbiota, have been validated in both *in vivo* and *in vitro* pancreatic cancer models to mediate poor prognosis by modulating the tumor microenvironment and suppressing host immune responses (Temel et al., 2023). Furthermore, studies reveal that lipopolysaccharides modulate CD4^+^ T cell secretion of TNF-α, IL-1β, and IL-8, inducing severe pancreatitis—a precursor event to pancreatic cancer. These findings suggest that metabolic products from the intratumoral microbiome not only directly chemically modify chemotherapy drug molecules to inactivate them but also indirectly interfere with drug activity by regulating tumor cell physiology and immune responses, collectively forming a critical component of chemotherapy resistance in pancreatic cancer.

### The role of intratumoral microbiome in regulating extracellular matrix deposition and remodeling

The pancreatic tumor microenvironment is highly fibrotic, and ECM deposition is widely recognized as a critical driver of chemoresistance (Feig et al., 2012; Sherman and Beatty, 2023). Pancreatic stellate cells dominate the tumor stroma, and the fibronectin secreted by these cells activates the ERK1/2 signaling pathway, thereby mediating gemcitabine resistance (Amrutkar et al., 2019). Although no direct evidence currently exists demonstrating that bacteria within pancreatic tumors regulate pancreatic stellate cells, studies have established that microbial components and metabolites can activate stromal cells in a similar manner in other fibrotic organs. For example, in liver cirrhosis, translocated bacteria activate kupffer cells via Toll-like receptor 4 (TLR4), which further stimulates hepatic stellate cells to differentiate into myofibroblasts and produce large amounts of collagen and ECM components (Maslennikov et al., 2023). *Bacillus subtilis*, a bacterium enriched in some fibrotic conditions, promotes fibrosis by activating stellate cells (Liu et al., 2022). Similarly, the gut microbial metabolite 3−indolepropionic acid enhances mitochondrial ROS production and triggers the p38/JNK pathway, thereby activating stellate cells and increasing ECM deposition (Yuan et al., 2023). Conversely, butyrate produced by certain microbiota can inhibit ECM deposition by targeting histone deacetylases in cancer−associated fibroblasts (Wang et al., 2024). By analogy, the intratumoral microbiome of pancreatic cancer likely uses similar mechanisms to regulate PSCs activation. These microbes may stimulate PSCs either directly through pathogen−associated molecular patterns (PAMPs) such as LPS, or indirectly via secreted metabolites, leading to excessive collagen deposition, increased stromal pressure, and compressed tumor vasculature. This dense ECM network severely impedes the delivery of chemotherapeutic agents such as gemcitabine into tumor tissues, resulting in insufficient drug exposure and subsequent chemoresistance. Therefore, regulation of ECM deposition and remodeling represents a vital mechanism through which the intratumoral microbiome compromises chemotherapy efficacy in pancreatic cancer.

## Pancreatic cancer treatment strategies targeting intratumoral microbiome and clinical advances

Intervention strategies targeting intratumoral microbiome have progressively transitioned from basic research to clinical application, which can be primarily categorized into three approaches:

### Probiotic engineering and delivery

Functional modification leveraging probiotics’ tumor-targeting properties achieves dual effects of microbiota regulation and immune activation. The LGG@Ga-poly functionalized system developed by Zhang Xianzheng’s team at Wuhan University—based on *Lactobacillus rhamnosus GG* (LGG)—features triple advantages through gallium-polyphenol network modification and chitosan nano-coating: First, gallium ions inhibit harmful bacteria without affecting LGG activity. Second, chitosan protects against gastrointestinal degradation. Third, it precisely targets tumors via the gut-pancreatic axis. Animal studies demonstrate that this system alone delays tumor initiation. When combined with gemcitabine, it achieves an 80% tumor inhibition rate (compared to only 15% with gemcitabine alone) and reduces chemotherapy-induced weight loss. When combined with α-PD-L1, it extends mouse survival to over 60 days (all mice in the α-PD-L1-only group died within 45 days) (Han et al., 2024).

### Synergistic effects of microbiome eradication and immunotherapy

Antibiotic depletion of harmful intratumoral microbiome can reverse immunosuppressive microenvironments and enhance the efficacy of immune checkpoint inhibitors. A Phase I clinical trial (NCT05462496) conducted by Mount Sinai Health System employed a sequential regimen of “chemotherapy + antibiotics + pembrolizumab”: For resectable pancreatic cancer patients, after chemotherapy, administer ciprofloxacin (500mg twice daily) and metronidazole (500mg three times daily) for 21 days, followed by pembrolizumab infusion. This aimed to activate T cells by clearing the microbiota (primary endpoint: 20% increase in activation rates of markers like HLA-DR and CD38), while also evaluating R0 resection rates and histological response rates. This study provided the first clinical validation of the feasibility of the “microbiota clearance - immune activation” synergistic mechanism (Wu et al., 2023).

### Fecal microbiota transplantation and metabolic regulation

Fecal microbiota transplantation (FMT) indirectly modulates intratumoral microbiome composition by reshaping the gut-pancreas axis microbial balance. Studies indicate that after receiving FMT from long-term pancreatic cancer survivors, recipient mice exhibit altered microbial diversity within pancreatic tumors. This effect slows tumor growth by inducing CD8^+^ T cell infiltration and increasing secretion of inflammatory factors IFN-γ and IL-2 (Riquelme et al., 2019). Combining FMT with 5-FU in pancreatic cancer treatment significantly inhibits tumor growth by regulating cytokines and increasing SCFA secretion (Li et al., 2025).

## Research challenges and future prospects

Although significant progress has been made in studying the intratumoral microbiome in pancreatic cancer treatment, numerous challenges remain. First, the technical standardization of microbiota research is inadequate. Variations exist in sample processing methods, sequencing platforms, and data analysis pipelines adopted in different studies, which limits the comparability of research results. It is therefore necessary to establish unified technical specifications and quality control standards. Second, the causal relationship between the intratumoral microbiome and pancreatic cancer remains incompletely elucidated. Most current studies employ correlation analyses, necessitating further validation of specific microbial functions through experiments such as germ-free animal models and microbiota transplantation. Additionally, substantial individual variability in microbiota, influenced by diet, lifestyle habits, underlying diseases, and other factors, poses challenges in developing personalized microbiota regulation strategies.

Future research may advance in the following directions: First, delve into the molecular mechanisms by which specific microbiota regulate chemotherapy resistance in pancreatic cancer, elucidating the interaction network among microorganisms, host cells, and chemotherapeutic agents to identify targets for targeted drug development. Second, develop precise microbiota detection technologies to assess intratumoral microbiome profiles through non-invasive methods such as liquid biopsies, enabling the identification of chemotherapy-sensitive populations and personalized treatment guidance. Third, explore multimodal combination therapies, such as integrating microbiota modulation with immune checkpoint inhibitors or targeted drugs to generate synergistic antitumor effects. Fourth, conducting large-scale clinical trials to validate the clinical efficacy and safety of microbiota modulation strategies, accelerating their translation into clinical applications.

Intratumoral microbiome has emerged as a novel therapeutic target in pancreatic cancer treatment, with its regulatory role in chemotherapy resistance offering new insights for improving patient outcomes. As research advances, therapeutic strategies targeting the intratumoral microbiome hold promise when integrated with conventional treatments, bringing renewed hope for pancreatic cancer patients.
